# Uveitis: basic concepts and differential diagnosis

**DOI:** 10.1186/1546-0096-12-S1-I4

**Published:** 2014-09-17

**Authors:** Clive Edelsten

**Affiliations:** 1 Ophthalmology, Great Ormond Street Hospital, London, UK

## 

Uveitis describes a variety of patterns of intraocular inflammation that may be linked with other localised ocular and orbital inflammations as well as extraocular disease. Expermental models of autimmune uveitis fail to reflect not only the clinical variety of ocular inflammatory disease but also the marked differences in pathology of human multi-system diseases that often result in very similar ocular disease.

There are marked differences in the epidemiology of paediatric ocular inflammatory diseases, compared to adults, and their rarity has needed a specific approach to both diagnosis and the evaluation of treatment.

The age -related differences in epidemiology partly relate to the earlier presentation of genetically driven inflammatory disorders as well as differences in clinical presentation of more commonly adult-onset inflammatory disorders. The recent advances in genetics require novel diagnostic pathways for ocular inflammatory disease as well as the revision of longstanding clinical descriptors.

Children's eyes react differently to inflammation, usually for the worse. Presentation is often late with established damage. This leads to a need for child-specific disease damage evaluation and clear separation of the complications subsequent to late presentation and those amenable to amelioration with effective immunosuppression. Reported outcomes need clear identification of the cohort characteristics. The limitations of existing outcome modelling is discussed as well as their relevance for cost-effective analysis of biologics in childhood uveitis.

## Disclosure of interest

None declared.

